# Planning guidance to limit hot food takeaways: Understanding the possible economic impacts

**DOI:** 10.1016/j.heliyon.2024.e38798

**Published:** 2024-10-05

**Authors:** Daniel Derbyshire, Antonieta Medina-Lara, Ben Amies-Cull, Michael Chang, Steven Cummins, Suzan Hassan, Matthew Keeble, Bochu Liu, Oliver Mytton, John Rahilly, Bea Savory, Claire Thompson, Martin White, Jean Adams, Thomas Burgoine, Richard Smith

**Affiliations:** a Public Health Economics Group, Department of Public Health and Sports Science, Faculty of Health and Life Sciences, University of Exeter, UK; b Nuffield Department of Population Health, University of Oxford, Oxford, UK; c MRC Epidemiology Unit, School of Clinical Medicine, University of Cambridge, UK; d Office for Health Improvement and Disparities, Department of Health and Social Care, UK; e Department of Public Health, Environments & Society, Faculty of Public Health & Policy, London School of Tropical Hygiene and Medicine, UK; f Great Ormond Street Institute of Child Health, University College London, UK; g School of Health and Social Work, University of Hertfordshire, UK; h Department of Urban Planning, College of Architecture and Urban Planning, Tongji University, Shanghai, China; i Department of Marketing, Faculty of Business and Economics, University of Antwerp, Antwerp, Belgium

**Keywords:** Hot food takeaways, England, Food environments, Planning policy, Economic impacts

## Abstract

Local and national policymakers are seeking innovative solutions to create healthier food environments around the world. Between 2009 and 2017, 35 local authorities across England (UK) adopted planning guidance designed to limit the proliferation of hot food takeaways near schools. Whilst these policies are intended to improve population health, they are also likely to have economic impacts. Often a decision to introduce such policies comes down to consideration of whether the short-term economic imperatives of allowing new takeaway outlets to open outweighs the potential long-term public health implications and associated economic consequences. These potential negative and positive economic impacts have not previously been clearly described and are summarised here. The aim of this paper is to provide an overview of the potential economic impacts of takeaway management zones. In particular, we present a Causal Loop Diagram (CLD) that outlines the possible economic impacts of takeaway management zones based on researcher knowledge of the interventions and the industry. Potential negative impacts fall across sectors and may include a loss of employment opportunities and reductions in local and national tax receipts, and may impact the economic vitality of local communities. In the longer term, there is the potential for positive impacts such as reductions in healthcare resource utilisation, social care expenditure and sickness-related absence from work. Part of a robust case would a better economic understanding, that would enable local authorities to improve understanding of the trade-offs associated with the policy, such as short-versus long-term, and business-related versus society-related health benefits and costs.

## 1 Introduction

Unhealthy diets are a product of the food environment – the social, political, and economic conditions within which people make their food purchasing and consumption decisions. They contribute to increased incidence of a wide range of non-communicable diseases, such as type 2 diabetes and hypertension. Unhealthy diet is also the primary cause of rising rates of obesity internationally [[1], [2], [3]]. In 2021, 23.4 % of 10–11 year olds and 25.9 % of adults in the UK were obese, with a further 14.3 % of 10–11 year olds and 37.9 % of adults overweight, and with the greatest propensity of both obese and overweight individuals falling broadly in more deprived areas [4].

Hot food takeaway outlets (hereafter, takeaways) have been identified as contributing to unhealthy food environments and unhealthy diets [[5], [6], [7]]. Food served by takeaways tends to be high in fat, salt, sugar and calories [8]. The number of takeaways in England has grown rapidly over the past decade from 24,725 ‘take away food shops and mobile food stands’ (i.e. Standard Industrial Classification (SIC07) 56.10/3) in 2012, to 38,400 in 2022, a 55 % growth [9], which reflects a changing food environment over time [10,11]. In England, there is evidence that takeaways are clustered around schools [12], especially in the most socioeconomically deprived areas [13]. Children in the most socioeconomically deprived areas consume more food from takeaways and have a higher Body Mass Index (BMI) compared to less deprived areas [14].

These trends have led to efforts in England to reduce proliferation of takeaways close to schools via the adoption of specific planning guidance to deny planning permission to new takeaways outlets in these vicinities. Takeaways are a specific use class within the English planning system and refer to outlets selling food intended primarily for consumption off the premises, regardless of the type or healthiness of food sold. Between 2009 and 2017, a total of 35 local authorities (LAs) adopted takeaway management zone planning guidance around schools [15]. However, the potential economic consequences of these policies has not been explored to our knowledge. In this paper, we discuss the potential positive and negative economic impacts that this class of intervention may have.

The aim of this paper is to provide an overview of the potential economic impacts of takeaway management zones. In particular, we present a Causal Loop Diagram (CLD) that outlines the possible economic impacts of takeaway management zones based on researcher knowledge of the interventions and the industry. The specific impact pathways are discussed to highlight the mechanisms underpinning some of the potential economic impacts.

## 2 Takeaway management zones

Takeaway management zones around schools can be used to deny planning permission or limit operating hours for new takeaway outlets within a certain distance of a school. The first of these policies was adopted by Waltham Forest Borough Council in 2009 [16]. These policies originally targeted planning applications for a Use Class Order (UCO; a particular usage classification within the English planning system) known as A5 that was specific to takeaways. More recently takeaways were given a ‘of its own kind’ use classification, which was introduced to make limiting takeaways easier by requiring change of use requests to be given full local consideration [17].

The precise implementation of these takeaway management zones around schools varies across the country. Of the 35 LAs that adopted this policy between 2009 and 2017, 20 % utilise ‘time management zones’ that restrict when takeaways can operate, as an alternative to refusing planning permission completely [15]. This involves, for example, new outlets not being allowed to open between 3pm and 5pm when children and young adults are leaving school. Most notably, 67 % adopted ‘town centre exempt’ zones meaning that takeaways would be permitted within the specified distance of a school if they were also in designated town centres or other retail sites [15].

There is evidence that takeaway management zones can affect the number of new takeaway outlets opening. For example, a study in Gateshead (England) showed a of significant reduction in new takeaways opening compared to a control group in the four years following adoption [18]. Conversely, a study of a takeaway management zone in Newcastle (England) did not show a significant impact on the number of new takeaway outlets in the three years following adoption [19]. A wider ranging aggregate-level study of 35 takeaway management zones found that the adoption of such policies led to a significant reduction in planning applications for new takeaway outlets [20]. Since there is some evidence that these takeaway management zones can have impacts on local retail composition by reducing the number of new takeaway outlets, it is important for local authorities to understand the potential economic impacts of this planning guidance.

## 3 Methods

Systems thinking is an approach that has been used to inform public health research that involves taking a ‘whole’ system approach that focuses on the complex relationship between various interdependent components of a given system [21]. Systems thinking approaches acknowledge the inherent complexity in many real world systems due to the interactions between the disparate aspects that make up the ‘whole’ system [22]. One method for utilising a systems thinking approach is the use of a causal loop diagram (CLD). CLDs are a qualitative system mapping method that provide a visual representation of the complexity of causal relationships between a set of relevant variables. Although CLDs are a long established approach in engineering and business management [23,24]; CLDs are now an increasingly popular tool within public health research and can be used to inform both policy and practice [25].

In the public health context, CLDs can be used to show causal relationships that demonstrate the impact pathways emerging from a particular intervention, including the potential for both commercial and behavioural reactions. Systems thinking approaches and CLDs have been used to investigate a wide range of public health issues including obesity prevention interventions [[26], [27], [28], [29]]. Whilst there are a variety of qualitative methods that can be used for creating CLDs, including stakeholder interviews and literature/document reviews, a commonly used approach is to create CLDs based on existing researcher knowledge and expertise [25]. Co-authors have experience using systems thinking approaches with respect to taxation of sugar-sweetened beverages [30,31] and have substantial experience of researching takeaways in general and takeaway management zones in particular. This includes the retail impacts of takeaway management zones [15,20], the public acceptability of takeaway management zones [32] and how both local government [16] and planning and public health professionals [33] use and experience takeaway management zones.

## 4 Economic impacts

**Fig. 1:**
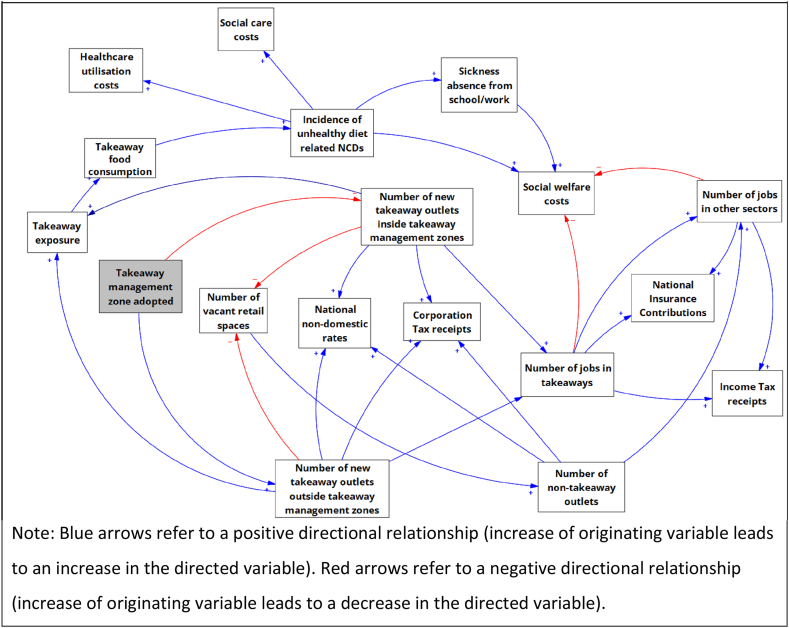
Causal loop diagram of the economic impacts of takeaway management zones Note: Blue arrows refer to a positive directional relationship (increase of originating variable leads to an increase in the directed variable). Red arrows refer to a negative directional relationship (increase of originating variable leads to a decrease in the directed variable). (For interpretation of the references to colour in this figure legend, the reader is referred to the Web version of this article.)

Despite a clear rationale in terms of improving local food environments and unhealthy diets in children, takeaway management zones can have a wide range of other potential economic impacts by affecting the composition of local retail in and around takeaway management zones. These are less well understood and explored within the literature, though there is evidence that restricting takeaways are an economic concern of policymakers [33,34]. Indeed, one of the most common variations is whether town centres are exempt from the planning guidance. This speaks to the concerns of policymakers with respect to hypothesised economic impacts, especially in more deprived areas where takeaways may make higher contributions to overall economic vitality. Indeed, planning authorities are required to ensure the economic vitality of town centres under the National Planning Policy Framework [35]. The CLD (Fig. 1) shows the mechanisms behind the primary hypothesised economic impacts of adopting takeaway management zones. In the following subsections, we discuss five potential channels which cover (1) businesses and vacant retail space, (2) government finances, (3) employment opportunities, (4) economic vitality and (5) health and social care-related spending. These five channels were determined based on an assessment of the qualitative evidence from previous work with policymakers and planning authorities regarding the impacts of takeaway management zones [16,33,36,37]. Following this, relevant variables for inclusion were identified using an iterative process beginning with the direct effect of takeaway management zones – that there will be fewer new takeaways opening [15]. From there, a combination of logical intuition and reference to the existing qualitative evidence were used to begin mapping out the impacts of takeaway management zones; such as higher numbers of new takeaways leading to higher numbers of new jobs in takeaways. The CLD went through two iterative rounds of review by the full project team to ensure the most relevant variables were included and appropriate relationships between variables had been identified before the CLD was finalised.

As an example of what can be seen in the CLD, adopting a takeaway management zone leads to a decrease in the number of new takeaway outlets within takeaway management zones (negative directional relationship). This in turn leads to a decrease in the number of jobs in takeaways (positive directional relationship) which leads to a decrease in income tax receipts and national insurance contributions (positive directional relationships) and a increase in social welfare costs (negative directional relationship).

### 4.1 Businesses and vacant retail spaces

There is evidence that takeaway management zones result in an increase in the number of new planning applications for takeaways being rejected [20] and a reduction in the number of new takeaways opening as a result [15]. In the case where a new takeaway is denied planning permission due to a takeaway management zone, unless the application is to build new premises, there will still be a retail space that could have some alternate use. The length of time that the retail space remains vacant and what the alternate use is or would be is a key consideration for estimating the magnitude of economic impacts in terms of employment opportunities and tax contributions. The potential for vacant retail spaces also represents a concern of policymakers [37]. In cases where the retail space quickly moves to an alternate usage, there will be a greater mitigation of the economic impacts than when a retail space remains vacant for a prolonged period. Some policies explicitly reference this situation and make dispensations for permitting takeaways when a retail space has been vacant for a prolonged period of time (e.g. six months in the case of Manchester) [38]. This also speaks to the opportunity cost with respect to employment created by takeaways and whether the fair comparator is no alternative employment (whilst a retail space is vacant) or employment in an alternative retail space use. Denying permission for new takeaways may leave retail spaces vacant for alternative uses that provide greater economic contributions than takeaways and this may offset the negative economic impact of fewer new takeaways and potentially generate net positive economic impacts.

A reduction in the number of takeaways inside takeaway management zones may be offset by an increase in the number of takeaways outside takeaway management zones. In particular, businesses may choose to instead locate themselves on the periphery of takeaway management zones (i.e. in the area just outside of the takeaway management zone that is not covered by the associated planning guidance). Increases in the number of takeaways outside of takeaway management zones has the potential to mitigate the economic impacts of takeaway management zones to the extent that the planning guidance may only encourage spatial relocation, though evidence suggests there may not be a significant takeaway displacement effect [15].

### 4.2 Government finances

Takeaways contribute to both local and national government finances. They contribute to the former through their tax payments in the form of business rates (commercial property taxes) to local authorities. Business rates are a substantial source of revenue for local authorities, accounting for 27 % of local authorities income in 2019/20 [39]. Local authorities are also responsible for providing social care, which was responsible for more than a quarter of local authority expenditure in 2019/20 [39]. Takeaway management zones have the potential to contribute to reductions in health and social care expenditures associated with unhealthy diets and non-communicable diseases such as type 2 diabetes and hypertension. There are also multiple forms of contribution to the national government finances made by takeaways. The food and drinks sold by takeaways is subject to a 20 % rate of Value Added Tax (VAT). Takeaways also provide contributions through their corporation tax payments to national government. The employment created by takeaways also impacts on government finances. This includes national insurance contributions, income tax receipts and reductions in out-of-work benefits associated with the employment opportunities created by takeaways. However, these impacts on employment related tax receipts (on both the employer- and employee-side) may be diminished by the higher prevalence of informal (unregistered) employment within the takeaways sector, since informal employment was never registered for tax purposes in the first place [40,41].

### 4.3 Employment opportunities

In 2021, there were 198,000 people employed in the takeaways sector in England (SIC07: 56/10.3 take away food shops and mobile food stands) [42]. The number of jobs in this sector has grown rapidly, too, by nearly 30 % between 2015 and 2021 (when there were 153,000 employees) [42]. The takeaways sector is therefore a non-trivial source of employment, responsible for 0.7 % of all employees in England, which is comparable to the number of people employed as solicitors (0.8 % of all employees) or in childcare provision (0.6 % of employees) [42]. Whilst takeaways do provide employment, there have been questions about whether these jobs are ‘Good Work’ (defined as work that is fair and decent with realistic scope for development and fulfilment) [43] and whether they represent desirable employment opportunities due to the associated sub-optimal pay and conditions [44,45]. For example, in 2021 more than half of employees (59 %) were part-time [42] and median hourly pay was £8.91 (SIC07: 56.10) [46]. The UK government defines low-paid employees as those earning less than two-thirds of national median hourly earnings in line with Organisation for Economic Co-operation and Development (OECD) definitions [47]. By this definition, low-pay employees are anyone earning below £9.85 (in 2021) and 44 % of accommodation and food service workers are low-paid, which is a higher proportion than in any other sector [48]. The takeaways sector also owes a portion of its success and growth to the rise of online delivery platforms and their use of zero hour contracts, which represent insecure, precarious under-employment [49].

The high proportion of part-time jobs and the flexibility of hours in the hot takeaways may appeal to those seeking flexibility or to certain groups of people e.g., students, parents or people with caring responsibilities. Further, people from ethnic minority backgrounds are more likely to be employed in the hospitality sector generally [50] and takeaways specifically [51]. However, the increased prevalence of informal employment [40] is associated with exploitative labour practices such as underpayment, long and unpredictable hours (60+ hours per week) and even forced labour [52].

### 4.4 Economic vitality

More broadly, takeaways are thought to contribute to the vitality of local high streets and to provide indirect increases in output and employment through economic multipliers. For the food and beverage service activities sector in 2019, the full time equivalent (FTE) employment effect was 22.7, meaning an increase in demand for food and beverage service activities of £1 million results in an additional 22.7 direct and indirect FTE employment [53]. This places food and beverage service activities in the top ten of more than 100 different sectors. The direct and indirect FTE employment multipliers are due to the establishment of supply chains [54]. This refers to the network of additional production relations that are required as a result of takeaways demand for inputs such as cooking equipment and packaging materials. However, this does not include further (induced) employment created by increases in footfall and economic vitality around takeaways, and so the employment contributions of takeaways are likely to be even higher. Any alternative usage of a retail space will also provide economic multipliers that may or may not compensate for the smaller number of new takeaways opening. Other factors which may have less directly tangible impacts but nonetheless affect local economies include litter, noise pollution, potential anti-social behaviour and traffic congestion typically associated with the existence of takeaways [36,37]. These ancillary impacts also correspond to other material planning considerations for planning authorities [16].

### 4.5 Health and social care related spending

There is potential for takeaway management zones to generate positive economic impacts in the long term through the reduction of co-morbidities and premature deaths associated with unhealthy diet and corresponding healthcare resource use. For example, the NHS costs associated with obesity in 2014/15 were estimated at £6.1 billion, with the wider costs estimated at £27 billion [55].

There are also further considerations around the social care costs associated with unhealthy diets and obesity. Social care refers to care outside of routine healthcare environments such as hospitals or doctors' practices, often taking place directly in the patient's home or within the community. These costs are also the direct responsibility of local authorities. Further, by generating reductions in unhealthy diets, takeaway management zones could lead to lower rates of sickness absence from work, directly increasing economic output. Sickness absence from work is estimated to be responsible for between £15 and £20 billion of lost economic output [56] and is associated with increased sickness related benefits payments. Reductions in sickness absence from school can indirectly impact on economic output by stopping parents from taking time off work for childcare purposes. Takeaway management zones therefore have the potential to generate substantial positive economic impacts, in particular by reducing health and social care resource costs and lost economic output associated with obesity and poor diet. Similarly, this planning guidance has the potential to increase long term human capital accumulation processes, by reducing missed educational opportunities due to sickness absence associated with unhealthy diet and obesity or other associated co-morbidities in children [57]. Targeting healthier diets therefore has the potential to create a future workforce that is both healthier and better educated, with all the associated economic benefits this entails.

## 5 Discussion

### 5.1 Policy implications

Characterising the economic contribution of takeaways to national and local economies is complex, especially with respect to economic vitality and the employment opportunities from takeaways as well as the potential long-term costs savings in health and social care. There can be a complex interdependence between the short-, medium- and long-term economic impacts of restricting the proliferation of takeaways. As such, a decision to introduce such policies often comes down to a consideration of whether the short-term direct economic imperatives of allowing new takeaway outlets to open outweighs the potential long-term public health implications. Without intervention by authorities, the commercial drivers underpinning the industry expansion will continue unabated and the number of takeaways – which are frequently co-located with school environments – will continue to grow. Without a proper assessment of the economic impacts, local authorities seeking to adopt takeaway management zones (or similar planning guidance in other locales) may face industry opposition based on unknown economic consequences. An approach to this could involve quantifying the various components of the economic impacts of takeaways using existing business micro-data (for example, to see how much takeaways spend on wages or specific taxes per year, on average). This would then be combined with estimates on the retail impacts of takeaway management zones of takeaway management zones [15]. The CLD presented in this paper can provide insights for both policymakers and academics wishing to investigate the potential economic impacts of takeaways. It is also worth noting that efforts to limit the proliferation of takeaways – either in general or close to schools – are not unique to the United Kingdom. Examples from around the world include Los Angeles (USA) [58], Canada [59], South Korea [60] and Australia [61]. While some of the specifics of taxation and spending arrangements may differ, the broad impacts will remain the same and therefore the CLD figure presented here may still be informative. Policymakers around the world seeking to improve food environments by limiting the proliferation of takeaways can adapt to their context the CLD presented here to inform their understanding of the mechanisms underpinning the potential economic impacts of their policies.

### 5.2 Strengths and limitations

This paper presents a novel CLD which combines the potential direct and indirect health and economic impacts of takeaway management zones around schools to give a comprehensive overview of the possible channels for positive and negative impacts from these policies. A lack of empirical data means that the relationships mapped in the CLD presented here have not been quantitatively tested and are instead based on a combination of logical intuition and qualitative evidence. However, it provides a framework for future research to attempt to quantify economically the impact of a new policy.

## 6 Conclusions

Takeaway management zones have the potential to generate both positive and negative economic impacts at both the local and national level. The causal loop diagram presented in this paper can help to inform policymakers and planning authorities about the possible interdependent relationships that contribute to the complexity of economic impacts deriving from takeaway management zones, and support researchers in establishing their impact.

## Funding

This study is funded by the National Institute for Health Research (NIHR) Public Health Research Programme (Project number: NIHR130597). The views expressed are those of the author(s) and not necessarily those of the NIHR or the Department of Health and Social Care. JR, MK, BL, MW, JA and TB were supported by the 10.13039/501100000265Medical Research Council (grant number MC_UU_00006/7). OM is supported by a UKRI Future Leaders Fellowship (MR/T041226/1).

## CRediT authorship contribution statement

**Daniel Derbyshire:** Writing – original draft, Visualization, Software, Methodology, Conceptualization. **Antonieta Medina-Lara:** Writing – review & editing, Supervision, Methodology, Conceptualization. **Ben Amies-Cull:** Writing – review & editing. **Michael Chang:** Writing – review & editing, Methodology, Funding acquisition. **Steven Cummins:** Writing – review & editing, Methodology, Funding acquisition. **Suzan Hassan:** Writing – review & editing. **Matthew Keeble:** Writing – review & editing. **Bochu Liu:** Writing – review & editing. **Oliver Mytton:** Writing – review & editing, Methodology, Funding acquisition. **John Rahilly:** Writing – review & editing. **Bea Savory:** Writing – review & editing. **Claire Thompson:** Writing – review & editing, Funding acquisition. **Martin White:** Writing – review & editing, Methodology, Funding acquisition. **Jean Adams:** Writing – review & editing, Methodology, Funding acquisition. **Thomas Burgoine:** Writing – review & editing, Funding acquisition, Conceptualization, Methodology. **Richard Smith:** Writing – review & editing, Supervision, Methodology, Funding acquisition, Conceptualization.

## Declaration of competing interest

The authors declare that they have no known competing financial interests or personal relationships that could have appeared to influence the work reported in this paper.
